# Lhasa childhood eye study: the rationale, methodology, and baseline data of a 5 year follow-up of school-based cohort study in the Tibetan plateau region of Southwest China

**DOI:** 10.1186/s12886-020-01522-w

**Published:** 2020-06-22

**Authors:** Weiwei Chen, Jing Fu, Zhaojun Meng, Lei Li, Han Su, Wei Dai, Yao Yao

**Affiliations:** 1 Beijing Tongren Eye Center, Beijing Tongren Hospital, Capital Medical University; Beijing Ophthalmology & Visual Sciences Key Laboratory, Beijing, 100730 China; 2 Beijing Institute of Ophthalmology, Beijing, China

**Keywords:** Methodology, Refraction error, Epidemiology, Children eye care, Public health

## Abstract

**Background:**

Tibetan Plateau is a highland area with special geographical location, time zone, and ethnic composition. We herein report the rationale, methodology and baseline data of the school-based childhood cohort study named Lhasa Childhood Eye Study (LCES), with the primary objective to pursue a comprehensive understanding on the longitudinal trends of refractive error as well as other ocular diseases and to address the differences between Tibetan Plateau and other parts of the world.

**Methods:**

Grade one students from primary schools in Lhasa were cluster randomly selected. They were examined and would be conducted with follow-up annually for 5 years. The examination procedures for LCES consisted of standardized ocular, systematic examinations, and questionnaires, identical to the Anyang Childhood Eye Study (ACES) conducted in central China.

**Results:**

One thousand nine hundred two Grade one students eligible for the LCES, 1856 (97.58%) participated in the study, with a mean age of 6.83 ± 0.46 years (range 5.89–10.32 years), and the proportions of male to be 53.02%. 1762 (94.93%) of the 1856 participants in the baseline exam were Tibetans. 1837 (98.98%) of the students examined had cycloplegic autorefraction performed. The numbers of hyperopia, emmetropia, myopia, and high myopia were 127 (6.91%), 1639 (89.22%), 71 (3.86%) and 3 (0.16%) respectively. Compared with ACES, students from LCES baseline had a younger age (*p* < 0.001), lower cycloplegic spherical equivalent (*p* < 0.001), similar myopia prevalence (*p* = 0.886), lower hyperopia prevalence (*p* < 0.001), and a higher emmetropia prevalence (*p* < 0.001).

**Conclusions:**

LCES was a school-based cohort study in Tibetan Plateau with a high baseline response rate. A higher emmetropic trend was found in LCES compared with ACES. Continuous documentation of this cohort might potentially provide useful reference information for the areas of China which was previously not well studied.

**Trial registration:**

The study has finished the clinical registration on Chinese Clinical Trial Registry. (ChiCTR1900026693).

## Strengths and limitations of this study

To the best of our knowledge, Lhasa Childhood Eye Study (LCES) is the first childhood cohort study in the Tibetan Plateau Region of Southwest China. Tibetan Plateau is a high-altitude plateau at high latitudes with specific lighting conditions, time zone, and ethnic composition.

### The major strengths of LCES include

1. Well-established and characterized cohort at baseline with high-quality ocular/systemic data.
2. High response rate (97.58%) and high cycloplegic percentage (99.84%) in the participants
3. The standardized protocol of most of the ocular examinations and questionnaires used in the LCES is the same as that of the Anyang Childhood Eye Study which is conducted in central China. This facilitates data pooling of the two studies to investigate ethnic and environmental factors related to childhood eye diseases.

### Limitations

1. Some of the risk factors might be potentially inaccurate due to the self-reported questionnaires from the students’ parents, even though the questionnaires used in the LCES are calibrated for cultural differences.
2. The portable equipment that can quantify exposures such as light and outdoor time and special parameters associated with plateau regions are not used in the baseline examination.

## Background

Visual impairment and visual disorders are important public health issues among children worldwide [1]. The most common causes of visual disorder in children are correctable or curable, such as refractive errors, amblyopia, and strabismus. But they could be serious conditions and lead to permanent visual impairment if left untreated [2, 3].

The understanding of the longitudinal trends consequently to get a clear knowledge of the eye care needs is the key point to prevent irreversible blindness in Children [4]. Many childhood cohort studies have reported the accurate observation of eye disease progression and provided precious clues for prevention and treatment needs among children, such as the Singapore Cohort Study of the Risk Factors for Myopia, the Orinda Longitudinal Study of Myopia, the Anyang Childhood Eye Study (ACES) and so on [5–9]. To our knowledge, there is a lack of longitudinal investigation on eye diseases of school-age children in the Tibetan plateau in China. Tibetan Plateau is a special area due to its geographical location, time zone, and ethnic composition. The composition and risk factors of children’s eye diseases in Tibet Plateau might be different from other regions of the world due to its special geographical and climatic environment, lifestyle, and cultural characteristics [10]. Therefore, we initiated and conducted a cohort study named Lhasa Childhood Eye Study (LCES).

We used the similar protocols of Anyang Childhood Eye Study (ACES) conducted in central China recently by another team of our hospital. This facilitated data pooling of the two studies to investigate ethnic and environmental factors related to myopia as well as other childhood eye diseases in children.

The overall objective of LCES was to determine the incidence and progression of major childhood eye diseases affecting Chinese Tibet Plateau children, such as myopia, amblyopia and strabismus and so on, to determine their relationship to traditional risk factors and explore a range of novel disease biomarkers, and to evaluate the eye care needs of these regions. Herein, we report the rationale and methodology of the LCES in detail and the key findings from the baseline.

## Methods

### Study design

The LCES is a school-based cohort study mainly designed to longitudinally observe the occurrence and development of different ocular diseases especially myopia in school-age children. The study adheres to the STROBE guidelines and the principles of the Declaration of Helsinki. The study has finished the clinical registration on *http://www.chictr.org.cn* (ChiCTR1900026693). Ethics committee approval was obtained from the Institutional Review Board of Beijing Tongren Hospital, Capital Medical University (TRECKY2019–146). Informed consent forms signed by the parents of all the participants were obtained prior to the start of the program. The LCES would be conducted from 2019 to 2024. We randomly selected a group of grade 1 students from the elementary schools in Lhasa and will follow them until they enter different middle schools.

### Criteria for participants and sample size considerations

Voluntary grade one students, living in Lhasa city for at least half a year and planning to continue to live there for at least 5 years, are legible for LCES. Individuals, suffering from mental illness or other medical conditions, were unable to cooperate with the baseline survey and follow-up would be excluded. According to the report from ACES which was conducted in central China recently, the 5-year cumulative incidence of myopia in school-age children was calculated to be 58.37% [7]. Considering that the incidence of myopia in Lhasa might be lower than in Anyang, we used the cumulative incidence of 40% to calculate the sample size. Assuming a design effect of 2.0, a tolerated error of 0.1 times the myopia incidence, and a loss of follow-up of 20%, 1382 grade one students would be needed. 27 out of the 28 elementary schools in Lhasa available joining the LCES were stratified into three levels based on the evaluation of local government. Finally, 1943 grade 1 students of 7 elementary schools were randomly sampled by stratified cluster sampling.

### Recruitment strategies

A coordination meeting was held by the local city government before the recruitment. Officials from city governments, the health, and the education departments of Lhasa summoned the principals of the selected schools and inform them of the importance and significance of the LCES. Local media such as television and newspapers were also used to publicize the study. The teachers in charge of each class were trained by the ophthalmologists from LCES to seek mutual understanding of the program and to ascertain the eligibility of each child. With the support of the principals and teachers, parents’ meetings were held to explain the importance and significance of the LCES. And then we invited them to participate in the program. Written informed consent forms were interpreted in detail and issued for signatures. The contact information of the Project Manager and Assistant Project Manager were given to facilitate communication between the study staff and the participants. Participants could withdraw from the study anytime. For those children and parents who didn’t agree to participate, at least three telephone calls were performed to seek their understanding. The children would be invited in-class-unit arranged by teachers in charge of a free check-up in the health examination station of LCES at an appointed time if eligibility criteria were fulfilled.

### Questionnaire survey

Only parent questionnaires were surveyed in LCES. Because most of the children were unable to fulfill the student questionnaires after we did a pilot study in one of the class chose. The questionnaires used in the LCES were mainly derived from the parent questionnaires used in ACES to facilitate comparisons between the two studies. We discussed with the representatives of local teachers, parents, and ophthalmologists to make sure the questionnaires culturally appropriate and linguistically accurate and no change needed. As reported in ACES the questionnaires’ reliability and validity were verified. In general, the questionnaires were designed to collect information about work, outdoor activity, date of birth and ophthalmic treatment, reading, living, as well as eating habits, and parents’ information such as refractive and socioeconomic status, education, pregnancy history, and medical records.

Since more than half of the parents were Tibetans and did not know Chinese, the questionnaires were translated and explained in detail by the trained teachers and the LCES staffs familiar with Tibetan and Chinese at the parents’ meeting. Difficulties about the questionnaire were resolved immediately during the meeting to make sure the results accurate. The fulfilled questionnaires were inspected by experienced staff of LCES according to the standardized protocol. Any equivocal points found from the finished questionnaires were clarified by interviewing the parents again. The questionnaire survey would be administered annually during the follow-up. The student questionnaires would be added from the one-year-follow-up on as the children might be able to understand and fulfill the questionnaires after one-year study.

### Examinations

**Fig. 1:**
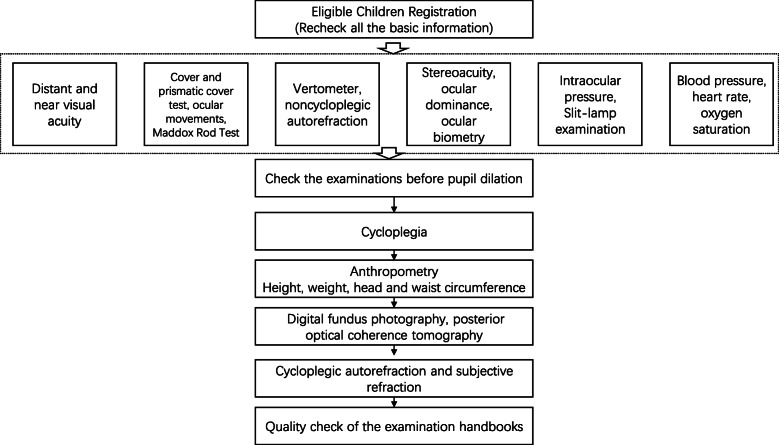
The procedure of ocular examinations for children in the Lhasa Childhood Eye Study. The examinations are composed of pre- and after-cycloplegia tests. Quality check will be executed before pupil dilation and after all examinations finished. Quality check includes making sure all examinations done before and after cycloplegia respectively, unusual value of the results

We performed all the examinations for the students in the health examination station of Lhasa Maternal and Child Health Care Center. The procedures were outlined in Fig. 1. Basic systematic examinations were composed of height, weight, body circumference, heart rate, blood pressure, oxygen saturation, etc. Standardized ocular examinations included distant, near and pin-hole visual acuity, identification of amblyopia and strabismus, ocular biometry, optical coherence tomography, retinal photography, cycloplegic autorefraction, intraocular pressure, stereo acuity, and ocular dominance. Examiners would perform the examinations according to the standard operation procedures (online supplementary).

### Quality control

The procedures of quality control were implemented throughout the study. The standard operating procedures were produced for staff training before data collection. All examinations were required to be performed following the procedures. For the fieldwork, two ophthalmologists (FJ, CWW) worked at the field site to ensure that the protocols were followed strictly. The examination handbook and questionnaires of every student were verified at least twice to evaluate integrity and precision. The students with missing data or erroneous data, would be asked to come back for ocular examinations or their parents would be contacted again for questionnaire. After verification of double data entry, 5% of the database was randomly selected to identify any inconsistency between the original documents and electronic records.

### Data entry and analytic plan for LCES

All the data were filled in forms and were independently entered into the database using Epidata software 3.1 (The Epidata Association, Odense, Denmark) by two individuals. The OCT results and Retinal photographs were stored in digital format for further post-processing and grading. Statistical analysis was performed using SAS software (version 9.4, SAS Inc., Cary, NC, USA). Age- and sex-specific prevalence estimates of myopia, amblyopia, strabismus and other ocular diseases, and the incidence of myopia would be calculated and analyzed. A general linear model (analysis of covariance) and a linear regression model would be used to evaluate the associations between risk factors and myopia at baseline. The Cox model would be used to analyze the risk factor of time to develop myopia during follow-up. Generalized estimating equation models would be used for eye-specific analyses.

## Results

### Study population and recruitment

**Fig. 2:**
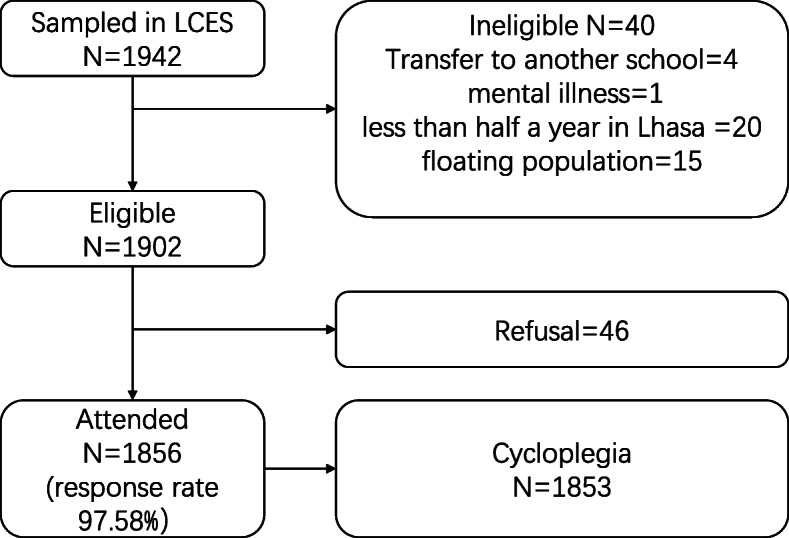
Lhasa childhood eye study (LCES) recruitment flow. Among the 1942 sampled students, 40 were ineligible according to the inclusion and exclusion criteria of LCES. Of the remaining 1902 eligible individuals, 1856 grade 1 students completed all the examinations with a response rate of 97.58%. 1853 (99.84%) of the examined students had cycloplegic autorefraction performed on both of their eyes

Recruitment details were shown in Fig. 2. Among the 1942 sampled students, 40 were ineligible for LCES according to the inclusion and exclusion criteria. Among the remaining 1902 eligible individuals, 46 did not attend the baseline exam, leaving 1856 grade one students who completed all the examinations during the period from 21st October to 18th November 2019, with an overall response rate of 97.58%; 97.14% for boys and 98.09% for girls.

### Characteristics of participants in LCES

For the 1856 participants in LCES, the mean age was 6.83 ± 0.46 years and 984 (53.02%) were males. Overall participant characteristics were shown in Table 1. 1762(94.93%) of the 1856 participants were Tibetan, while 85 (4.58%) were Han, 9 (0.49%) were other nationalities.

**Table 1:** Characteristics of participants who attended baseline LCES (*N* = 1856)

Characteristics	Mean ± SD	Median	Range
Age, years	6.83 ± 0.46	6.78	5.89–10.32
Gender, male, *n* (%)	984 (53.02%)		
Ethnic categories, *n* (%)			
Tibetan	1762 (94.93%)		
Han	85 (4.58%)		
Others	9 (0.49%)		
Height, cm	120.55 ± 5.52	120.00	103.00–168.00
Weight, kg	22.96 ± 3.69	22.00	12.00–45.00
BMI, kg/m^2^	15.74 ± 1.80	15.45	9.23–27.47
IOP, mm Hg	16.02 ± 2.73	16.00	8.00–26.00

Data presented are mean ± SD or frequency (%), where appropriate

*BMI* body mass index, *IOP* intraocular pressure, *LCES* Lhasa Childhood Eye Study

### Refractive status

Among the 1856 grade one students examined, 1853 (99.84%) had cycloplegic autorefraction performed on both of their eyes. Cycloplegic spherical equivalent had a median of + 1.13 D (range − 9.00D to + 8.25 D) (Table 2). The prevalence rates of myopia and high myopia were 3.94 and 0.16%, respectively. The prevalence rates of emmetropia and hyperopia were 89.15 and 6.91%, respectively. The prevalence was based on data from the right eyes. A significant difference was found in the overall comparison of the refractive status between LCES and ACES using Fisher’s accurate test. Comparisons of refractive status in detail were shown in Table 2.

**Table 2:** Comparison of refractive status between LCES and ACES

Parameters	LCES			ACES			Statistic value	*P*
	n (%)	Median	Mean ± SD	n (%)	Median	Mean ± SD		
Age, years	1856	6.78	6.83 ± 0.46	2893		7.1 ± 0.5	T = 18.73	*p* < 0.001^a^
Cycloplegic SE	1853	+ 1.13D	+ 1.07 ± 0.92D	2749	+ 1.50D	+ 1.44 ± 1.05D	T = 12.78	*p* < 0.001^a^
Hyperopia^c^	128 (6.91)	+ 2.38	+ 2.78 ± 1.03	64 (2.3)				*p* < 0.001^b^
Emmetropia	1652 (89.15)	+ 1.13	+ 1.05 ± 0.49	2003 (72.9)				*p* < 0.001^b^
Myopia^c^	73 (3.94)	−1.00	−1.53 ± 1.49	106 (3.9)				*P* = 0.886^b^
High myopia^c^	3 (0.16)	−6.25	−7.16 ± 1.59	2 (0.1)				*P* = 0.398^b^

*D* diopter, *SD* standard deviation, *LCES* Lhasa Childhood Eye Study, *ACES* Anyang Childhood Eye Study

^a^The Independent-Samples T-test was used to compare the age and cycloplegic SE between LCES and ACES; ^b^The Bonferroni method was used to calibrate the test level for the pairwise comparisons of the refractive states; ^c^Hyperopia, myopia and high myopia were defined as spherical equivalent≥ + 2.00D, ≤ − 0.50D, and ≤ − 6.00D, respectively (based on data from the right eyes)

## Discussion

To the best of our knowledge, no other school-based childhood cohort study was conducted in the Tibetan plateau. LCES is a meaningful childhood epidemiology study. First, Tibetan Plateau is a high-altitude plateau at high latitudes with specific lighting conditions. Second, the bedtime and rising time of people there are potentially influenced when using Greenwich Mean Time + 8 while living in the Greenwich Mean Time + 10 zone. Third, more than 90% of the residents are Tibetans with particular living habits and cultural habits. The special food, architectural, and nomadic cultures might cause differences in the myopia development or prevalence of other eye diseases [10]. These characteristics make the sampled cohort have special living environment, lighting conditions, living habits, and cultural habits, and ensure findings from LCES to be an important complement to other population studies in China, as well as the world [11–13].

Lhasa, the capital of the Tibet Autonomous Region of the People’s Republic of China, was selected as the study area due to its relatively stable demographic structure. Lhasa is located in the middle of the Tibetan Plateau, with an average altitude of 3650 m [14]. Lhasa has three districts and five counties. The three urban districts of Lhasa selected for LCES have 28 elementary schools with about 47,000 primary school students and more than 8000 first-grade students every year. The enrollment rate of primary-school-age children is 99.7%, mainly Tibetan children. 94.93% of the participants in LCES were Tibetans. There were 27 out of 28 elementary schools participated in LCES sampling and the response rate was 97.58%. These advantages ensured the samples in the LCES representative of the urban Tibetan plateau region of southwest China and higher participation rates during the follow-up.

The major strengths of LCES include a well-established and characterized cohort at baseline, high-quality ocular/ systemic data, high response rate, and good comparability with studies in other parts of China and the world. The standardized protocol of most of the ocular examinations and questionnaires used in the LCES is the same as that of the ACES which is conducted in central China [7]. This facilitates data pooling of the two studies to investigate ethnic and environmental factors related to myopia. These advantages made it possible to achieve specific aims of LCES: 1) to describe the figure and development of refractive status, ocular biometry, retinal thickness parameters by OCT, retinal-vascular related parameters among plateau children from elementary school over a period of 5 years; 2) to document the prevalence, incidence as well as risk factors of myopia and other childhood ocular diseases such as amblyopia, strabismus and so on, and then to summarize the eye care demands in children in the Tibetan plateau region of southwest China so as to provide clues for intervention; 3) to compare regional and ethnic differences in childhood ocular disease incidence, progression, and associated risk factors.

In the presented report, we described the refractive status of the LCES baseline with an extremely high response rate of cycloplegic test (99.84%). The refractive status (+ 1.13 D) and the prevalence of myopia (3.94%) of the grade 1 children in the LCES were comparable with children in northern, southern, central and western mainland China (1.0–1.36D) reported previously as well as children in the Baltimore Pediatric Eye Disease Study and Multi-ethnic Pediatric Eye Disease Study [15–21]. However, the prevalence of myopia was much lower than Chinese children living in Taiwan, Hong Kong, Singapore, and Canada but much higher than that of the USA [17, 22–24].

The students from LCES younger than ACES, had a lower cycloplegic spherical equivalent, similar myopia prevalence, lower hyperopia prevalence, and a higher emmetropia prevalence. One of the reasons for the higher emmetropic trend might be lifestyle changes with time (LCES 2019 vs ACES 2011). The factors associated with these differences were meaningful and would be analyzed in subsequent reports.

However, there are still some limitations to the present study. Firstly, some of the risk factors might be potentially inaccurate due to the self-reported questionnaires from the students’ parents, even though the questionnaires used in the LCES were calibrated for cultural differences. Secondly, some portable equipment that can quantify exposures such as light and outdoor time and special parameters associated with plateau regions were not used in the baseline examination. We are planning to add them during follow-up of the LCES children.

## Conclusion

In summary, the LCES is a longitudinal study on childhood ocular diseases in plateau regions of Chinese children. A higher emmetropic trend occurred in the students of Lhasa compared with students in central China. Continuous documentation of the 5-year follow-up from LCES is expected to have an important, positive clinical impact. The results from this special population may potentially provide useful reference information for the areas of China which were previously not well studied.

## Data Availability

The datasets used and/or analyzed during the current study are available from the corresponding author on reasonable request.

## Abbreviations

LCES: Lhasa Childhood Eye Study
ACES: Anyang childhood Eye Study
