# Correction to: Lhasa childhood eye study: the rationale, methodology, and baseline data of a 5 year follow-up of school-based cohort study in the Tibetan plateau region of Southwest China

**DOI:** 10.1186/s12886-020-01572-0

**Published:** 2020-07-31

**Authors:** Weiwei Chen, Jing Fu, Zhaojun Meng, Lei Li, Han Su, Wei Dai, Yao Yao

**Affiliations:** 1 Beijing Tongren Eye Center, Beijing Tongren Hospital, Capital Medical University; Beijing Ophthalmology & Visual Sciences Key Laboratory, Beijing, 100730 China; 2 Beijing Institute of Ophthalmology, Beijing, China

**Correction to: BMC Ophthalmol 20, 250 (2020)**

**https://doi.org/10.1186/s12886-020-01522-w**

After publication of our article [1] the authors have notified us of an error in Table 2. The corrected Table 2 is presented below (modifications marked in red).

**Table 2:**
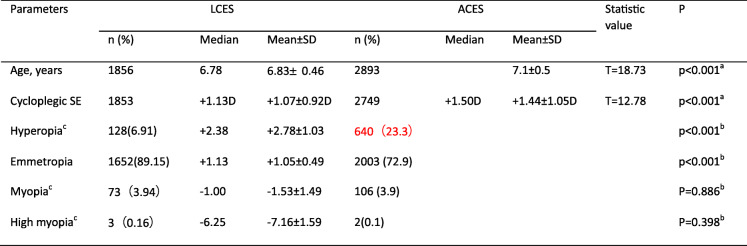
Comparison of refractive status between LCES and ACES

*D* diopter, *SD* standard deviation, *LCES* Lhasa Childhood Eye Study, *ACES* Anyang Childhood Eye Study

^a^The Independent-Samples T-test was used to compare the age and cycloplegic SE between LCES and ACES; ^b^ The Bonferroni method was used to calibrate the test level for the pairwise comparisons of the refractive states; ^c^Hyperopia, myopia and high myopia were defined as spherical equivalent≥ + 2.00D, ≤ − 0.50D, and ≤ − 6.00D, respectively (based on data from the right eyes)
